# Grasping the experience of the other from an interview: Self-transposition in use

**DOI:** 10.3402/qhw.v8i0.20634

**Published:** 2013-08-23

**Authors:** Sanne Angel

**Affiliations:** Department of Nursing Science, Institute of Public Health, Aarhus, Denmark

**Keywords:** Heidegger, interview, partner, rehabilitation, self-transposition, spinal cord injury

## Abstract

This article describes a part of the interview process that is never usually reported. Listening to what people say is the key to increasing our knowledge of human existences. Procuring knowledge about human experience is much more challenging. Although good sources on how to prepare and conduct an interview exist, the process of the interviewer's perception of the interviewee's message and meaning is less examined. Beyond the role of eliciting the data, the researcher endeavours to reproduce the interviewee's narration and not the voice of the researcher. By illustrating the process during the interview, further transparency and thereby validity may be achieved. To exemplify this, the perception of the interviewer is explored, and here Heidegger's work on self-transposition has proved to be helpful.

This article explores the interviewer's perception of the participant's situation. The example includes the expressions of one man's experiences, which is part of a study of seven partners to newly spinal cord-injured persons. In the study (Angel & Buus 2011), we learned about the partners’ suffering despite an unharmed body, and how the initial shock transformed into increased vulnerability over the years. This was brought on by the changes to everyday life such as increased physical and mental strain, numerous and new responsibilities and tasks that at times were experienced as overwhelming. The extent to which they mourned their loss, the change of lifestyle, and the lack of self-realization varied. It was the long-term outlook that seemed to be decisive for the partners’ ability to endure the present situation and keep their spirits up: did it seem likely that things would improve to the satisfaction of both parties, so the partners could achieve some kind of balance between the necessary everyday tasks and their need for freedom and self-realization. To elucidate these experiences, interview was the method of data collection. The intention of this article is to illuminate the process of the interviewer's perception during the interview. In a detailed example, reflections elaborate the interviewer's perception of what the interviewee relates.

## Background

Procuring knowledge about human experiences involves challenges. Listening to what people say is the key to increasing our knowledge of human existences. To achieve this, the quest for the interviewer is to elicit the interviewee's experiences. The pursuit of knowledge may even go far beyond what the interviewee is aware of before the interview. Especially in a discipline like nursing, the interaction with and the support for patients creates a need to know more about perspectives and other issues, which the patient may not have ascribed meaning to or simply has not reflected on due to a debilitating state of consciousness, illness, or crisis. In situations like these, not all events are reflected upon. Klawonn (2003) explains this as pre-reflective experiences, which release an amount of experiences that do not call for reflection. This may be in line with Sartre's (1994) theory that only the experiences that differ from the known will result in cognitive recognition, but also experiences that do not make an impact because the person lacks the capacity to receive and perceive the impression. In both situations, the experience will not be put into words and cause no reflection. In research, this means that the interviewer may ask questions that the interviewee has not reflected on prior to the interview. The reflection invokes the interviewee to elaborate on the events and experience expressing them. This necessitates knowing the terminology for them, and that the interviewer's and the interviewee's terms mean the same, which the interviewer must constantly ensure during the interview. Alongside the general communication issues, the interviewer must endeavour to understand a person who perhaps does not even understand him/herself or has difficulties confessing his/her experiences to the interviewer or even to him/herself. Hence, the words do not necessarily convey the patient's experience that he/she is seeking to disseminate to the interviewer. Understanding the spoken word of the other and consequently his/her experiences and situation is therefore a challenge that can more or less be outweighed on the basis of a context. Impressions do not evolve from verbal expressions alone. Not only does the interviewer listen to the words in the search of their meaning. In the quest to familiarize him/herself with the situation the interviewee is trying to give him/her access to, the interviewer is deeply absorbed and focused on the interviewee's expressions. Thus, the meaning is sought in the span of the sentences and the way they are spoken in a given context.

Good sources on how to prepare and conduct an interview are available (e.g., Kvale, 2007). In qualitative research, the interviewer's active role is acknowledged as a necessity in eliciting the data. Kvale's (2007) work is an example of an explanation of the entire research process that deals with interviews. This more general approach to interviewing is supposed to be linked to the varied qualitative traditions. In addition, a good deal of the decision-making is left to the interviewer to decide which conduct that matches the recommendation to the actual research question and the actual participant. This calls for an iterative approach to the conduct. The interviewer's interaction with the interviewee during the process depends on the interviewer's ability to elicit the required illumination of the research question. This builds on reflections in action, and the conduct of which is explained very abstractly, perhaps due to the difficulty in being precise in this field of iteration. Focusing on the interviewer's perception is a core element in understanding the interviewer's endeavour to give the interviewee's voice. The interviewer's ability to understand more than the spoken word is central to being a skilled, trustworthy interlocutor, being able to ask questions that reveal abundant answers. Regardless, the core of these skills is that the conduct is determined by the specific situation. Nevertheless, more insight may be gained by exploring the conduct in retrospective.

In most everyday communication, the exchange of information is carried out with questions and answers until the participants feel that they have expressed themselves to the full. This does not require any deeper reflection, being the way people communicate, understand, and relate. This ability is explained by Dilthey who said “For everything in which the human spirit has been objectified contains in itself something which is common to the I and the thou” (Dilthey, 1977, pp. 126–127). This is the foundation for mutual understanding. Of course, people misunderstand each other from time to time or simply do not understand what the other person is trying to say. But the general interaction between people builds on our ability to get an impression of what the other person wants to express. At the same time, we are well aware of the tendency to read one's own feelings and experiences into the interpretation of the other. The challenge is to keep in mind that the first-person perspective reveals the second-person perspective due to interpretation and understanding but may only mirror the first person's own perspective.

In the research interview, the aim is to achieve data about the studied matter, and not the interviewer's perspective on the matter. It is suggested that this can be promoted by addressing the interviewer's preconceptions. The interviewer's preparation for listening to the interviewee includes determining how to elicit the necessary data. Sometimes, this implies asking very specific questions in order to get specific answers. But, when we seek the experiences of others, the challenge is to assume an open attitude in order that one's own perceptions do not hinder this. The extent and level of this openness is an on-going discussion, to which Husserl's seven kinds of epochês (Embre, 2011) are in contrast to Ricoeur's more pragmatic request of being as open as possible, although he regards pre-conception as an absolute condition (Ricoeur, 1983). Both leave it up to the interviewer to operationalize the openness.

The validation of data can be done by examining the questions and answers in the transcribed interview. Kvale (2007) suggests this as a way of interpreting whether the interviewee's story is directed by the interviewer's answers. But what happens during the interviewer's reflective process between the questions and answers. The question is, can transparency be enhanced by descriptions of how the interviewer's subjective experience (the second-person perspective of the interviewee's experience) turn into profound insight in and understanding of the other's experience (the first-person perspective of the interviewee)?

## Purpose

The purpose of this article is to exemplify how transparency in interpretation can be increased during the perception process in an interview situation. This transparency is first and foremost the interviewer's own awareness of how the interpretation came about. By reporting his/her reflections, they provide transparency to the person reading the data analysis on how the researcher interpreted the data.

## Methodological consideration

The illustration in this article builds on an investigation and description of the impressions that constituted my perception of the interviewee. As an example, I have used a quote that stems from one interview in a study of partners’ experiences after their partners had suffered a spinal cord injury (Angel & Buus 2011). The main source and focus for the interpretation was a telephonic interview that took place 2 years after the accident. However, the context for interpretation also included sources from previous contacts with the participant, Kent and his wife, Kate. Thus, I had an insight from an interview with Kent 1 year previously, and with Kate, a recent interview and five interviews and seven field observations during the first year. In order to convey that I did not jump to conclusions, I will try to elaborate on how my interpretation was elicited. Kent's story is outlined on the basis of all the collected data and retold. According to Polkinghorne (1995), this is also a way of providing solid data. The researchers’ narratives may put the interviewee's sometimes sporadic narratives into perspective and thereby provide a more readable material. I then explain and reflect on the analysis of the interview with Kent 2 years after the accident. This outlines one specific quote that seems fruitful in an illustration of the process of perception. On this basis, I recall how the interview with Kent affected me: my feelings, bodily reaction, and thoughts.

## Kent's story

Kent is a man in his 40s. He has been married to Kate for more than 15 years. Together, they have two children, the eldest, a teenage boy and the youngest, a girl at primary school. Before the accident, they practiced a traditional division of tasks. Kent and Kate both worked. Kent worked longer hours than Kate, while she in turn took care of the household and the tasks related to their children and family life. During their leisure time, they enjoyed an active sporting life together with their children.

Kent is still working full time. The hours away from his family give him the energy to cope with the family's present situation. He says that when Kate phones him at work and he only hears her voice, he sometimes forgets all about the injury. Before the accident, he could focus fully on his job but he now plays a major role in the household. Kate still takes responsibility for the children and their home, but she needs him to do a lot of the things that she used to do. The outdoor activities they used to enjoy in their spare time are not possible for the family any more. At home, Kate now just sits in her chair watching the tasks pile up despite her assistant's help, and waits for Kent to come home. When he does, she needs his help to do these tasks, to go to the toilet during the evening and the night, to fetch the things she cannot reach, to support her doing her exercises and to help her into bed. Kent is tired, and in dire need of rest. He could not recall having seen one full-length TV programme since Kate's accident. Kate's dependency makes her feel as though she is in prison and Kent is the one who can set her free from time to time. Being needed to this extent makes Kent almost feel suffocated, although he still wants to fulfill Kate's needs.

## The analysis of the interview

The research question in the study of the partners’ experiences (Angel & Buus 2011) was “what is it like being the partner of a person who had suffered a spinal cord injury.” From the analysis of the interview using Ricoeur's (1976, 2008) three analytical steps: first, the naïve reading to reach an initial perception of the whole text. The second step is to examine the text's structures and through this structural analysis, sentence by sentence, to verify or falsify the very first understanding. Finally, the third step serves to qualify the most significant interpretation among possible interpretations on behalf of the two first steps.

Kent's story occasioned the understanding that being a partner could be experienced like being trapped with no way out and still managing somehow. Despite his hopes that Kate would improve, he did not mention at any point an expectation of a changed situation. Instead, he just hoped that he would have more energy when the summer came, and looked forward to feeling relieved when he had adapted to the situation. Kent's feeble hope motivated him however to search for new rehabilitation programmes, although he knew that they might not improve his wife's prognosis. His circumstances were characterized by love and compassion, but also a completely different life full of new responsibilities. In Kent's case, leaving him exhausted and distressed and with no prospect for Kate improving. However, I have learned from other participants in the case of Ib from Angel & Buus' (2011) study that despite the same characteristics his experience of the present and expectations for the future were almost complete opposites. He had expected the worst-case scenario, and so every little bit of progress was experienced as a cadeau, and even though being there for his wife took up all of his time, he expected that this would change in the future. He told me this after his wife's condition had improved considerably, although this did not change anything about Kent's experiences. I will refrain from explaining away the different experiences due to different personalities, different relationships and different professional care at the time of the injury, the spouses’ different outcome, levels of dependency and reactions. Not that this does not interest me, but even though there are possible explanations, it does not change the fact that being a partner to a severely injured person can be experienced as Kent did. After spending a very long time trying to interpret his story and understand his situation, I realized that he had told me in just one sentence what I found to be the very essence of the analysis. I will therefore use this sentence to illustrate the analysis in depth. In Danish he said “Det går da, det skal jo gå, det er da lidt træls, det hele, men, sådan er det” (Well …, I'm OK, I have to be—it's a bit of a grind though, but that's how it is).

## The interviewer's perception of the interviewee

To investigate my impressions, I recalled how the interview with Kent affected me; my feelings, bodily reaction, and thoughts. I interviewed Kent by telephone, because he said that time was short and a phone call would be less demanding. Kent spoke in a low, tired voice. The sound was monotonous and this intonation hit me so hard that my own intonation changed from open interest to grave acknowledgement of his difficult situation and a deep concern for him. I experienced the feeling of being trapped although it was Kent who was trapped, and not me. Acute sorrow filled my body with a heavy sensation. Just a moment before, I had not been aware of my body. I pictured his home with all the undone tasks, underlining my feeling of the fatigue, he had expressed. An urge to ease his situation made me realize that this would most probably be impossible. My ability to imagine another future disappeared, leaving me to be in this unbearable situation together with him, just enduring it. It pinpointed the meaning of “it's going ok though” making me sense that it most likely was not ok at all. This was supported by his lack of expectations in relation to Kate's physical improvement. Instead, his hope addressed expectation of the coming summer's positive impact, and his own adaptation.

When Kent said in a tired voice “Well …, I'm OK” (“det går da”) it meant that the days just passed one after the other, and he had somehow managed. I realized that 1 year had actually gone by since the first time I spoke to him. Already at that time, 1 year after the accident, he felt completely exhausted. This is more of an assessment of time, looking back on the time that has just gone by without him noticing, and the problems and all the things that arose had been solved even though they seemed impossible to overcome at the time. He continued “but it's a bit of a grind, though” (“men det er da lidt træls det hele”). The word “grind” grasps the meaning of the Danish word “træls” which stems from a word “træl” meaning slave. In present-day Danish, “træls” implies being bored, but this is a more profound feeling of sadness, frustration, and a desire to escape from the situation. Describing life as a bit of a grind implies a lack of good times: a kind of treadmill, lumbering on doing the same things everyday with no prospect of change. Also, implying both the repetitive commonplace tasks with no endpoints and no rewards; tasks that normally did not take that much time and energy, and now they are taking over everything. His use of the word “a bit” contrasts with his tired voice. Does he say “a bit” because this is the only way he can find to open up and talk about an existence on the borderline of what he can manage? Still, he cannot and will not give up. He cannot give up because of his duty to his wife, and will not give up because of compassion for her. He expresses this in the words: “Well … I'm OK” “I have to be” (“det skal jo gå”). There is no way out; his beloved wife suddenly finding herself in a situation she cannot handle, their children doing their best to cope with the situation and manage what they could on their own and being supportive to the best of their ability. His situation seemed to have embodied itself in him. Talking about what he usually enjoyed doing, he replied that he did not feel up to it at the moment. He simply did not have the energy to pursue what he found agreeable before. He is so fatigued that he is unable to find the mental and physical strength. He is also unsure whether he will be able to carry on. These words expressed the feelings he awoke in me; a feeling of sadness, despair and fatigue—and seeing no way out. He concludes: “but that's how it is” (“men, sådan er det”). This is a kind of resignation; he cannot do anything about it and nobody else can at the moment. However, he is not completely defeated yet, although not quite sure whether he is getting close. He hoped that Kate would improve a little more, so that she would be less dependent on him, not only in order to give him some spare time but also so that Kate can regain her appetite for her life and be able to do something on her own instead of always having to ask her helpers or him.

All this was the sum of: the tone of his voice, my perception during the entire interview, the vibrations he awoke in me, and the insight I had from an interview with Kent 1 year previously, a recent interview with Kate, and five interviews and seven field observations of her during the first year.

## Discussion

So, how can I justify that I have revealed the essence of Kent's experience? Can an interviewer's impression of the participants’ experience be acknowledged as sound research or will it be suspected of being the interviewer's subjective fiction? This is not a question I can answer. But I can try to explain why this should be acknowledged as such.

According to Heidegger (1962, 2007), the discourse in itself implies being-with-one-another in learning about the other's being and world. Heidegger writes about communication (*Mitteilung*): “... through it a co-state-of-mind (*Mitbefindlichkeit*) gets shared, and so does the understanding of with-being” (Heidegger, 1962, p. 205, 2007, p. 191). This means that during a conversation one person can get access to the other's experiences. Not only due to the speech and silences, but also due to sharing the moment, which in itself measures the mood (*Befindlichkeit*). Reflecting on the interview situation, I will try to describe how I experienced this. In order to understand the depth of Kent's experiences, I tried to restrain my pre-understanding and linger with the situation that Kent was trying to describe. I tried to envisage it as I listened to Kent's words and their meaning and to capture the situation as Kent experienced it. In doing this, my perspective shifted from my own first-person perspective to a second-person perspective allowing Kent's words and mood to sink in. Like tunes being played on a piano resound with depth and fullness in the wooden body of the piano, Kent's expressions resonated in me. Resonate understood as a second-person capacity of awareness that enables a perception of the first person's mood (Churchill, 2012). It seems as if our being-with-one-another made my first-person perspective mirror his first-person perspective mood. As we talked, my mood shifted totally from being satisfied and aware of myself to a feeling of being trapped with no way out. Heidegger (1953, 1962, 2007) points at the understanding being-with-one-another as both referring to the attitude and the needed pre-conception to understand what is being experienced.^1^ I dwell on the impressions I had received, and put my interpretations on hold, refraining from drawing any sort of conclusion, not even provisional ones. I let the fatigue and hopelessness in his voice resonate in me, not really wanting to proceed to the stage where I would ascribe the tiredness and hopelessness to the experiences of Kent. This can be compared with the study on seduction by Lingis (2012), describing how the impression of the other's tone triggers an emotion and how the listener ascribes this emotion to the other. Although I identified the fatigue in his voice, I did not jump to the conclusion that Kent was tired. I dwelt with the impression and tried to find out why it was conveyed. This is an example of disclosing an impression, which is often grasped as a whole.

Having an open attitude meant that own perspectives and own experiences moved into the background in order to perceive the experiences that the other is wording. Keeping own experiences in the background only means letting the other's world stay in the foreground. Heidegger (1995) puts it like this “We ourselves being precisely ourselves and only in this way first bringing about the possibility of ourselves being able to go along with the other being while remaining *other* with respect to it” (Heidegger, 1995, pp. 202–203). This means that the interviewer's experiences and receptivity are working to perceive the other in his situation through the spoken words, the silence, the sighs, the laughter, the voice, and the mood that accompanies it. This corresponds to what Heidegger reveals in his study of the discourse (Heidegger, 1962, Section 34; Heidegger, 2007, Section 34).

According to Merleau-Ponty's (1964) theory of perception, this is possible, and reiterating Husserl's words, that even though one cannot be the other person, and one can only grasp the essence of what is constituted in the other, a person is still able to grasp this essence because she is a person herself (Merleau-Ponty, 1964). According to Merleau-Ponty, this means that even though I cannot read Kent's mind, I can get an idea of what he experiences through a perception of him and his expressions (Merleau-Ponty, 1964). This means that although I do not have access to the other's intentional life, I have access to an outline of it. However, grasping this outline of the essence makes it possible to perceive the central matter of the other's experience. This essence of human experience mirrors the meaning it gives me, and I can share in the other person's attunement. Perhaps one could say that the essence comes down to the emotional tone. Heidegger (1995) and Dilthey (1977) before him have used expressions like *Sichversetzen* and *Sichhineinversetzen*. Translated into English the term is self-transposition. Heidegger says “Transposing oneself into this being means going along with what it is and how it is. Such going-along-with means directly learning how it is with this being discovering what it is like to be this being” (Heidegger, 1995, p. 202). This is based on the assumption of the ability to put oneself into the other's situation (Heidegger, 1995). The distinctive thing about the successful research interview is that the other person agrees to disclose personal experiences, opens up and consents to the transposition of the interviewer. This “consent” is what makes the self-transposition possible according to Heidegger (1995).

In putting oneself into the other's situation, we stay ourself and this gives us an opportunity to understand what the other person understands with a surplus of understanding from our own perspective. Heidegger (1995) says: “Perhaps in doing so we may even see right into the nature of the other being more essentially and more incisively than that being could possible do by itself” (p. 202). We may then elicit plausible descriptions of how the other's experiences were perceived. In the analysis above, I divided the eidetic of the impression into parts by locating my description, which probably neither came from Kent's words alone, nor entirely my imagination—but something in between. For example the sadness: he did not express in words how sad he was, but in his way of speaking and phrasing the words supported the impression of being sad to the extreme. But one could ask: where did my intuition of Kent begin? Was it his choice of words? It was not his bodily expression understood as body language because I interviewed him by telephone. Perhaps it was the tone of his voice that was so intense that it made my heart, stomach and legs feel so heavy? But his tone did more than attune. It also conjured up a picture of Kent, I could imagine him in front of me; sitting with an almost expressionless face, his shoulders drooping. His words corresponded to his tone of voice and created a total impression. As he began to speak I was captivated by his story. His words and tone of voice resulted in an eidetic of his situation; of how tired he was, of how he kept up his hopes for Kate, of their home with all the tasks left undone. These objects were all part of my perception of Kent's world. Another thing was a strong sense of the difficulties of the deadlocked situation he was facing along with my knowledge that the chance of improvement was almost nonexistent. I became worried that he would not be able to cope with the situation, which made me consider his tone of voice; and even though I experienced something close to hopelessness, this was not quite despair and surrender. I verified this by remembering how some of his statements had an ambiance of strength and being in control. Churchill (2012) elaborates on Husserls’ thoughts on empathizing perception, saying about the second-person perspective that “It is a lived bodily experience in which a ‘felt sense’ of the other's ‘interiority’ (namely, my resonating with the other's intentionality) is given to me spontaneously, …” (Churchill, 2012, p. 3) in order to be even more precise he expresses this moment of attunement like “… I feel present to the other's soul” (Churchill, 2012, p. 3). This example of attunement from hearing the other's voice adds to the well-known face-to-face, gaze-to-gaze and Churchill's (2012) illustration of attunement with a letter as the medium.

Concluding when the perception as a whole is decomposed, it opened for an insight into the process of self-transposition that reveals the very basis of involvement. This implies an attunement from the second-person perspective until the first-person perspective. By describing the interviewer's reflective process during the interview, transparency can be enhanced. The description of the self-transposition reveals the interplay between the spoken sentences, the situation, the vocal and bodily expressions, and the emotions, thoughts, and reflections elicited in the interviewer. Thus, disclosing elements of self-transposition may be a useful tool in research justifying how the interviewer elicits human experiences.

## Footnotes

1 I have chosen to paraphrase Heidegger's quote “Als verstehendes In-der-Welt-sein mit den Anderen ist es dem Mitdasein und ihm selbst ‘hörig’ und in dieser Hörigkeit zugehörig” (1953, p. 163) because a footnote indicates difficulties in the English translation (1962, p. 206).
